# Remediation of Manganese-Contaminated Coal-Mine Water Using Bio-Sorption and Bio-Oxidation by the Microalga *Pediastrum duplex* (AARLG060): A Laboratory-Scale Feasibility Study

**DOI:** 10.3389/fmicb.2019.02605

**Published:** 2019-11-12

**Authors:** Jakkapong Thongpitak, Jeeraporn Pekkoh, Chayakorn Pumas

**Affiliations:** ^1^Ph.D. Degree Program in Environmental Science, Environmental Science Research Center, Faculty of Science, Chiang Mai University, Chiang Mai, Thailand; ^2^Center of Excellence in Bioresources for Agriculture, Industry and Medicine, Department of Biology, Faculty of Science, Chiang Mai University, Chiang Mai, Thailand

**Keywords:** microalgae, photosynthesis, bio-oxidation, adsorption, kinetics

## Abstract

Acidification occurs as a result of acid mine drainage after the oxidative weathering of metal sulfides. The acidic condition corrodes other toxic elements from the soil and becomes distributed around the operating site. Although coal mines go through a process of rehabilitation, water samples in the rehabilitated reservoir still reveal high concentrations of certain metals, for example, manganese (Mn). Both living and non-living biomass substances were used in Mn remediation. However, using non-living biomass as a sorbent may be inappropriate for the purposes of upscaling in high-volume water bodies. Thus, living microalga, *Pediastrum duplex* AARLG060, has become of significant interest for this type of application. The Mn remediation of microalga was performed by biosorption and bio-oxidation. The aim of this study was to evaluate the potential of microalgal Mn remediation of the water obtained from a rehabilitated coal-mine reservoir. The equilibrium and isotherm values of the remediation process were also studied. The microalga was used to remediate Mn in water under three different water conditions, including filtrated water obtained from the rehabilitated site, non-filtrated water that was sterilized with an autoclave, and non-treated water. Remediation was performed by culturing microalga with modified medium consisting of N, P, C, and Mg nutrients. The remediated Mn concentration present in the cultures was detected by atomic absorption spectroscopy. The precipitated Mn was collected as a result of bio-oxidation, and EDTA was used to wash Mn from the biomass. This was designated as an adsorption result. Characterization of biosorption was evaluated by employing the Langmuir and Freundlich models. The results demonstrated that all treatments of living microalga could support Mn bio-oxidation. The Mn remediation was successfully performed at over 97% in every treatment. The adsorption characteristics revealed a close similarity to the Langmuir isotherm of monolayer adsorption. The scanning electron microscope–energy dispersive spectroscopy (SEM–EDS) indicated precipitation of Mn oxide on the cell surface, while transmission electron microscopy (TEM) revealed that the nanoparticles of Mn were scattered mainly in the chloroplast and throughout the vacuoles of the cells.

## Introduction

Acidification can occur as a result of the lignite coal mining process. Runoff and drainage from these types of operating sites reveal acidic conditions. Additionally, many corrosive heavy metals are released into natural water bodies that are located nearby (Mohamed, 2001). Although many coal mining operations have been closed and the sites have been rehabilitated, the water in some of the surrounding areas is still acidic and may remain contaminated by heavy metals (Balintova et al., 2012). One of these problematic heavy metals is manganese (Mn) (Phillips et al., 1995). Mn is one of the trace elements needed by living organisms. This element functions as a catalyst in various enzymatic reactions. However, high levels of Mn can induce neurotoxicity in humans (Dobson et al., 2004; Niemiec and Wiśniowska-Kielian, 2015). According to Thailand’s Industrial Effluent Standard Guidance, the concentration of Mn in effluents should not exceed 5 mg/L.

Manganese remediation techniques have been developed in the last few decades. The traditional method of Mn remediation is chemical oxidization, which involves changing soluble Mn to its insoluble form. The precipitated MnO_2_ will then be removed by membrane filtration (Silva et al., 2012). However, chemical Mn oxidation requires a specific pH level of over 7.5. This process requires a large amount of chemical agents when being applied to larger bodies of water. Biological treatments offer an alternative method for Mn remediation (Chaput et al., 2015). Non-living microbial biomass of bacteria, fungi, and algae have been used to remediate Mn. Non-living aquatic plants that are adsorbent materials derived from suitable biomass can be used for the effective removal and recovery of wastewater containing low heavy metal ion concentrations. However, these plants would not be suitable for large-scale use in high-volume water bodies. The amount of adsorbent saturated to the limit of the sorption sites and the rate of increase for the degree of heavy metal removal was not proportional to the increase in the adsorbent. This may be attributed to the interference between the binding sites at higher metal concentrations (Bunluesin et al., 2007). Meanwhile living microbials are considered more appropriate than non-living microbials in large-scale laboratory applications. In addition, these microbials can remove heavy metals under a wide range of pH values and temperatures. Moreover, they are capable of growth under low nutrient conditions as well as in standing water (Eweis et al., 1998; Rangsayatorn et al., 2002). Living green microalgae are made up of microorganisms that show promise and potential in Mn remediation. This is because living microalgae could remove Mn via both bio-sorption and bio-oxidation. Biosorption is a combination of the adsorption and absorption processes of Mn. In this process, Mn ion is adsorbed onto the surface of the cell walls by functional groups such as carboxyl (COOH), phosphate (${\text{PO}}_{4}^{3-}$), and hydroxyl (OH^–^) (Kaplan, 2013). Shortly afterward, the absorption process may occur. Some soluble Mn ion is transferred into the cell, fixes with a metalloid protein, and accumulates within the vacuole of the microalgae (Mehta and Gaur, 2005; Kaplan, 2013). Another remediation process involves bio-oxidation. This occurs when microalgae photosynthesize and take up carbon dioxide from the water. This process produces oxygen as a byproduct and indirectly assists in raising pH levels, both of which involves oxidizing soluble Mn to MnO_2_ (Richardson and Stolzenbach, 1995).

Manganese remediation using living microalga *Pediastrum duplex* AARLG060 has been reported elsewhere. A simple and low-cost medium that could promote microalgae growth at elevated pH levels and support Mn remediation has been developed (Thongpitak et al., 2018). However, the study that accomplished this was conducted using synthetic wastewater. Thus, this study will focus on the potential of using living microalga for the remediation of soluble Mn in natural wastewater.

A lignite coal mine situated in the northern part of Thailand (500292E, 1966086N) was selected as the source of natural wastewater for this study. Mining operations at this coal mine have been suspended since the end of 2008 due to the depletion of coal reserves. Since 2014, this coal mine has been going through a period of rehabilitation. The operating site now appears as a large reservoir with water storage capacity of approximately 4.66 × 10^6^ m^3^. Information pertaining to the quality of the water in this reservoir is presented in Table 1. However, the remediation process using natural wastewater can be challenging due to a number of uncontrollable factors such as the activities of other microalgae or other microorganisms. Consequently, the feasibility of Mn remediation of living microalgae using water obtained from the rehabilitated site should firstly be performed on a laboratory scale.

**TABLE 1:** Some physico-chemical parameters of the rehabilitated lignite coal-mine in northern Thailand.

**Parameters**	**Units**	**Low**	**High**	**Average**
pH		4.15	4.31	4.23 ± 0.113
Suspended solids (SS)	mg/L	ND	9.95	9.95
Total solid	mg/L	861	895	878 ± 24.04
Arsenic	mg/L	ND	<0.0010	ND
Cadmium (Cd)	mg/L	0.00185	0.00196	0.001905 ± 0.000078
Calcium (Ca)	mg/L	142	164	153 ± 15.55
Copper (Cu)	mg/L	0.0109	0.0117	0.0113 ± 0.000565
Magnesium (Mg)	mg/L	30.18	39.74	34.96 ± 6.76
Mn (Mn)	mg/L	8.6	19.40	14.00 ± 7.64
Mercury (Hg)	mg/L	<0.0010	ND	ND
Sulfate (as ${\text{SO}}_{4}^{2-}$)	mg/L	460	535	497.5 ± 53.03
Total hardness (as CaCO_3_)	mg/L	540	720	630 ± 127.27
Turbidity	NTU	1.25	1.32	1.285 ± 0.05
Zinc (Zn)	mg/L	0.291	0.324	0.3075 ± 0.02
Iron (Fe)	mg/L	0.09	0.133	0.1115 ± 0.03
Lead (Pb)	mg/L	ND	ND	ND
Electrical conductivity	μS/cm	1021	1035	1028 ± 9.90

The aims of this experiment were focused on the ability of the living microalga *P. duplex* AARLG060 on Mn biosorption and bio-oxidation using actual water that had been obtained from a rehabilitated lignite coal mining site. Equilibrium and isotherm modeling were also studied.

## Materials and Methods

### Alga Culture

Green microalga *P. duplex* AARLG060 was obtained from the Applied Algal Research Laboratory (AARL), Department of Biology, Faculty of Science, Chiang Mai University. The microalga strain was isolated from a natural water reservoir located in the northern region of Thailand (Prasertsin et al., 2014). This microalga was chosen because a previous study indicated that it could be used to remediate and generate the oxidation of Mn at high Mn concentrations (Thongpitak et al., 2018). An axenic culture of microalgal stock was maintained in Jaworski’s medium (JM) at 25°C under continuous shaking and illumination with light emitting diode (72.51 μE m^–2^ s^–1^). The *P. duplex* AARLG060 biomass was collected by centrifugation at 4000 rpm for 20 min. The microalgal cells were then washed twice with modified medium consisting of N P C and Mg (Thongpitak et al., 2018).

### Natural Contaminated Wastewater

The sampling site was located at a rehabilitated reservoir located in the northern part of Thailand (500292E, 1966086N). Water samples were collected from the surface of the reservoir. The samples were then kept in a 20-L bottle of high-density polyethylene (HDPE) at 4°C before being used.

### Batch Experiment

#### Microalga Growth

Batch cultures of living microalga *P. duplex* AARLG060 were cultivated in natural contaminated wastewater obtained from the rehabilitated reservoir. The modified medium that had been optimized for low cost and high Mn remediation from the previous study was used. It consisted of NaNO_3_ (0.09438 g/L), KH_2_PO_4_ (0.02606 g/L), CaHCO_3_ (0.0159 g/L), and MgSO_4_.7H_2_O (0.0500 g/L) (Thongpitak et al., 2018). Each treatment was grown in triplicate at ambient temperatures using LED illumination (111.81 μE m^–2^ s^–1^). The light conditions were set to simulate high light intensity for large-scale outdoor remediation. Microalgal growth was determined daily by the measurement of optical density at 665 nm (OD_665_) using a spectrophotometer (genesis 20, ThermoScientific Fisher, United States). The pH value was measured using a pH meter (starter3100, OHAUS, United States). Initial microalga optical density was recorded at 665 nm (OD_665_) and adjusted to 0.2 for each treatment. Additionally, 300 mL of microalga was cultivated in 500 mL Erlenmeyer flasks. The cultures were manually shaken three times per day to confirm that all alga cells had been suspended. Manual agitation was employed to prevent dissolved oxygen from agitation, which could interfere with Mn oxidation in the microalgal photosynthesis.

#### Mn Remediation of Natural Wastewater Obtained From a Rehabilitated Reservoir

Manganese concentrations in this experiment were assigned as the lowest and the highest concentrations recorded of water collected from the rehabilitated reservoir at 9 and 20 mg/L, respectively. To investigate the effects of the biotic factors in natural wastewater, an experiment was conducted employing three treatments that involved low Mn non-filtration (LM-NF), high Mn filtration with non-sterilization (HM-F-NS), and high Mn filtration that had been sterilized by autoclave (HM-F-S).

#### Determination of Mn Concentration

**FIGURE 1:**
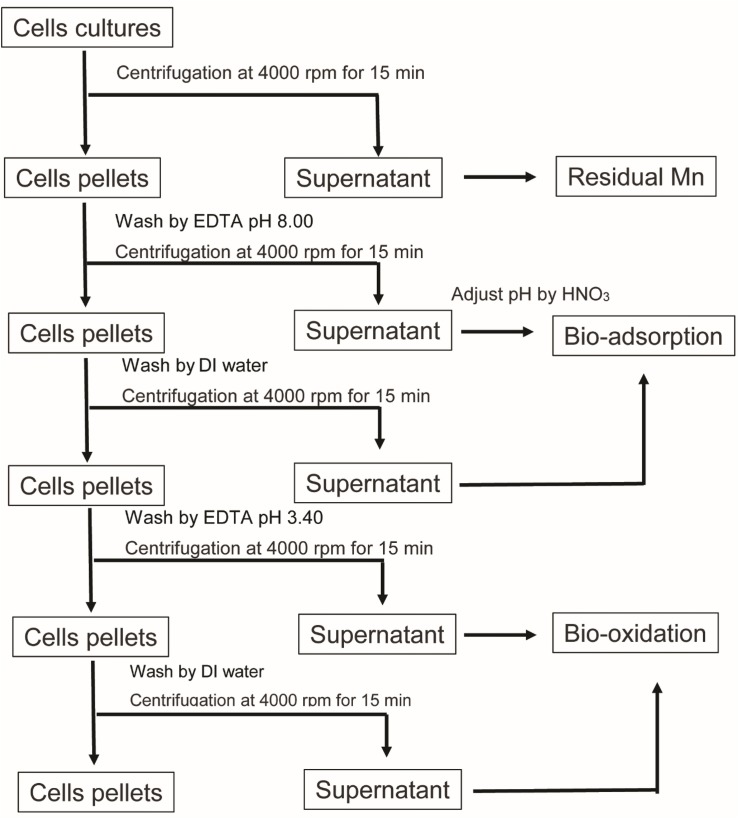
Schematic diagram representing the Mn content analysis in each portion of the remediation.

To investigate the biosorption and bio-oxidation abilities in terms of the Mn remediation of the microalga, Mn concentrations were determined according to the APHA method using the SP-AA 4000 spectrum instrument Atomic Absorption Spectrometer. After the cultivation process, 10 mL of cells was harvested for determination of Mn content at 0, 1, 3, 6, 9, 12, 24, 72, and 144 h. The Mn concentrations in each portion were collected and assigned as is shown in Figure 1. Firstly, the cultures were centrifuged, then the supernatants were collected and were assigned as residual Mn. Afterward, the cells were washed with 10 mL of 1 mM EDTA at a pH value of 8.00. The cells were then washed with deionized water. The supernatants obtained from EDTA washing and the deionized water washing steps were collected and analyzed for Mn adsorption. Finally, the amounts of precipitated Mn oxidation that remained with the cell pellets were dissolved by 10 mL of 1 mM EDTA at a pH of 3.40. They were then analyzed using the same method applied to the Mn concentrations in the supernatant (Webster et al., 2011).

### Mn Remediation Efficiency

Adsorption capacity (*q*_*e*_), which was used to explain the amount of adsorbed Mn on the cellular surface as unit per milligram of biomass, was calculated at the level of equilibrium for mg/mg using the following equation:

(1) $$q_{e}=\left(\frac{C_{i}-C_{e}}{m}\right)\,V$$

Mn remediation can be presented by percentage using the following equation:

(2) $$Mnremediation\left(\%\right)=\left(\frac{C_{i}-C_{e}}{{\text{C}}_{i}}\right)\times 100$$

where *C*_*i*_ and *C*_*e*_ are the initial and equilibrium concentrations of Mn (mg/L), respectively. Meanwhile, *m* is the representative of the amount of mg of the biomass and *V* represents the volume in liters of microalgal culture used in the experiment. The microalgal biomass was collected after cells were washed with EDTA and deionized water. Cells pellets were dried in a hot air oven at 60°C for 2 days, or until the dry weight was stable. The amount of dry biomass used in the sorption calculation is shown in the Supplementary Material. Additionally, *q*_*e*_ was compared in total Mn remediation and Mn adsorption. Total Mn remediation calculated using *C*_*e*_ obtained from the residual Mn, while Mn adsorption was determined by using *C*_*e*_ obtained from EDTA wash at a pH value of 8.0 (Figure 1; Kenduzler and Turker, 2002).

### Adsorption Kinetics

Adsorption kinetics, which are described as the rate of adsorption based on the solid capacity and the adsorption process governed by the active site of the microalga surface of the cell, were determined using the pseudo first- and second-order models. The pseudo first order was expressed as follows:

(3) $$q_{t}=q_{e}\,\left(1-\text{exp}\,\left(-k_{1}\,t\right)\right)$$

where *q*_*t*_ and *q*_*e*_ (mg/mg) represent the amount of metals adsorbed at time *t* (h) and at a level of equilibrium per biosorbent (mg/mg), respectively. Additionally, *k*_1_ represents the pseudo first-order rate constant (1/h).

The kinetic characteristics may be determined by the pseudo second order. The pseudo second order is expressed as follows:

(4) $$q_{t}=\frac{q_{e\,}^{2}\,k_{2}\,t}{1+q_{e}\,k_{2}\,t}$$

where *k*_2_ represents the equilibrium rate constant for the pseudo second-order sorption (mg/mg⋅h) and *q*_*e*_ (mg/mg) represents the amount of metals adsorbed at time *t* (h).

### Isotherm Adsorption

The level of isotherm adsorption that can reveal the adsorption capacity of the adsorbent is representative of the amount of adsorbate that is taken up by the mass of the adsorbent. The levels of Langmuir and Freundlich isotherm adsorption were analyzed in most studies. Langmuir isotherm adsorption is described as the monolayer adsorption characteristics on the adsorbent. The non-linear model is expressed as follows:

(5) $$q_{\text{e}}=\frac{q_{m}\,k_{L}\,C_{e}}{1+k_{L}\,C_{e}}$$

where *q*_*m*_ (mg/mg) is the maximum sorbate taken up by the adsorbent and *k*_*L*_ is the Langmuir coefficient *C*_*e*_ that represents the heavy metal concentration in the solute. The plot of 1/*C*_*e*_ versus 1/*q*_*e*_ shows a slope with a value of the intercept/slope and an intercept value of 1/*q*_*m*_.

The Freundlich model is used to represent the heterogeneous sorbent system. The non-linear value of this model can be expressed as follows:

(6) $$q_{e}=k_{F}\,{\left(C_{e}\right)}^{1/n}$$

where *k*_*F*_ and *n* are Freundlich constants and the plot of log *q*_*e*_ versus log *C*_*e*_ has a slope with a value of 1/*n* and an intercept magnitude of log *k*_*F*_.

### Characterization of Bio-Mn Oxide

Manganese oxide that formed on living microalga *P. duplex* AARLG060 was harvested via centrifugation at 4000 rpm and fixed with 2.5% of glutaraldehyde in 0.1 M of phosphate buffer at a pH of 8.00 overnight at 4°C. The samples were then washed with phosphate buffer at a pH of 8.00. Shortly afterward, the samples of microalgae were dehydrated with ethanol at a concentration of 50–100%. In the next step, microalga were mounted on stubs and thereafter were gold-sputtered (Michalak et al., 2014). Bio-Mn oxide formation was observed and photographed using a scanning electron microscope (SEM) JEOL-5410LV SEM. The SEM was equipped with an Oxford INCA energy-dispersive spectroscopy (EDS) system to capture the distribution of the elemental composition on the surface of the microalga cell wall. The X-ray spectrum of each microalga was obtained along with the given microelement composition.

### Effect of Mn on Microalgal Ultrastructure

To investigate the effect of Mn on the ultrastructure of microalga, transmission electron microscopy (TEM) was used. In the procedure that was proposed by Kumar and Filippi (2016), the microalga cells were fixed with 2.5% of glutaraldehyde in 0.1 M of phosphate buffer at a pH of 8.00, as was similar to the SEM process. In the next step, microalga samples were washed with phosphate buffer at a pH value of 8.00 and fixed with 1% of osmium tetroxide (OsO_4_) solution in the phosphate buffer at a pH of 8.00 for 2 h. After being washed with the buffer, the samples were dehydrated with ethanol at concentrations of 50–100%. Shortly afterward, they were embedded with a 2:1, 1:1, and 1:2 mix of propylene oxide:resin for 1 h and the samples were fixed for 24 h in pure resin. All sample were polymerized at 60°C for 24 h. Finally, ultrathin sections were mounted on cupper grids and evaluated in a JEOL JEM-2200 FS TEM at 200 kV.

## Results

### *Pediastrum duplex* AARLG060 Growth and Mn Remediation

**FIGURE 2:**
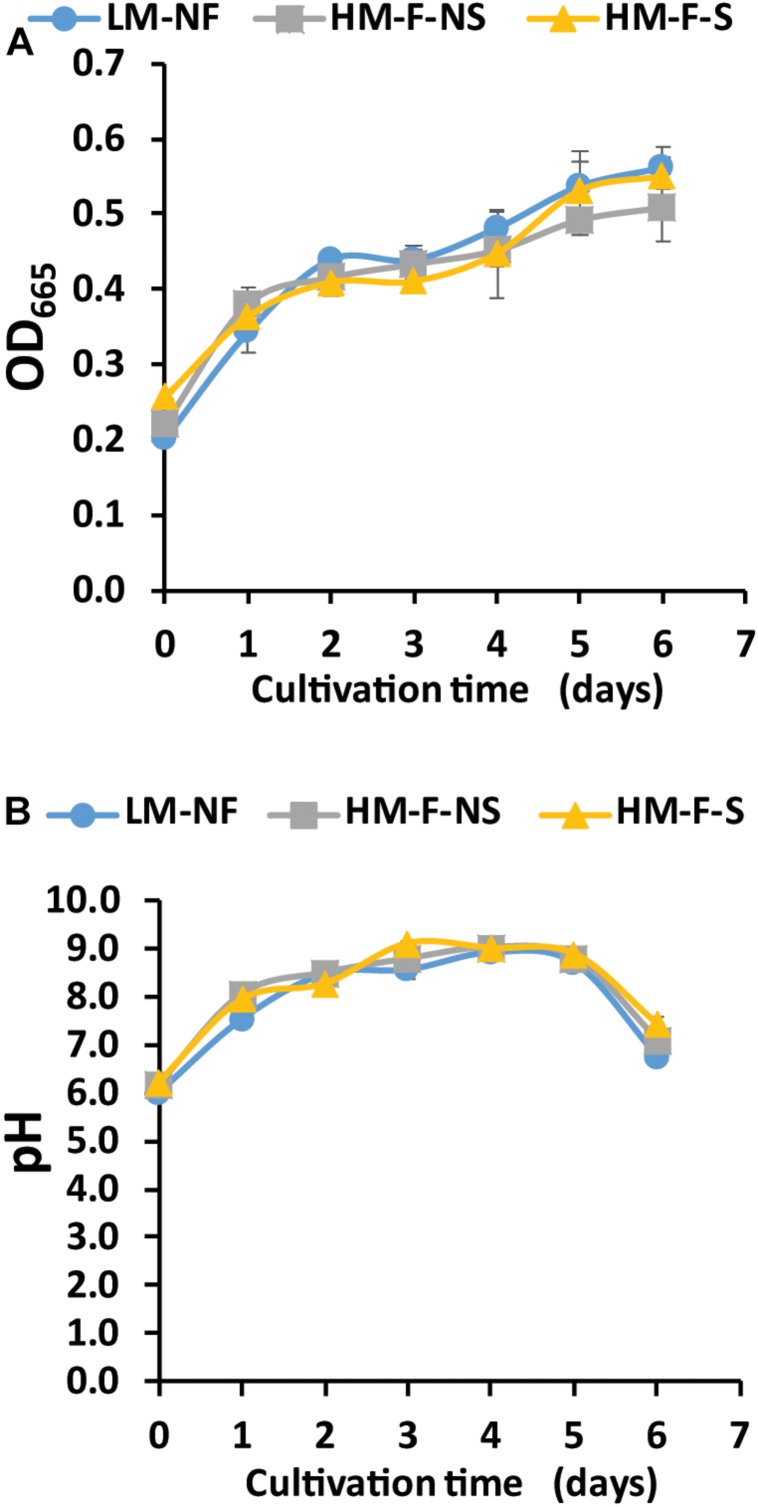
Optical density **(A)** and pH value **(B)** of *Pediastrum duplex* AARLG060 cultivated with Mn remediation using biosorption and bio-oxidation processes. LM-NF, low Mn non-filter; HM-F-NS, high Mn filter non-sterile; and HM-F-S, high Mn filter sterile.

The modified medium consisting of N, P, C, and Mg was used to investigate the growth of microalga in Mn remediation. In this experiment, microalga was cultivated with modified medium in LM-NF, HM-F-NS, and HM-F-S. Microalga growth, as indicated by optical density (OD_665_), revealed that the fast growth of OD_665_ increased from 0.2 to 0.4 over 2 days for all of the treatments (Figure 2A). This growth also enhanced the pH level. The pH level of the modified medium started at approximately 6.0 and slightly increased under alkaline conditions (Figure 2B). The pH levels of LM-NF, HM-F-NS, and HM-F-S were over 8.0 from the 2nd day until the 5th day of cultivation, which could support Mn bio-oxidation by microalgae.

**FIGURE 3:**
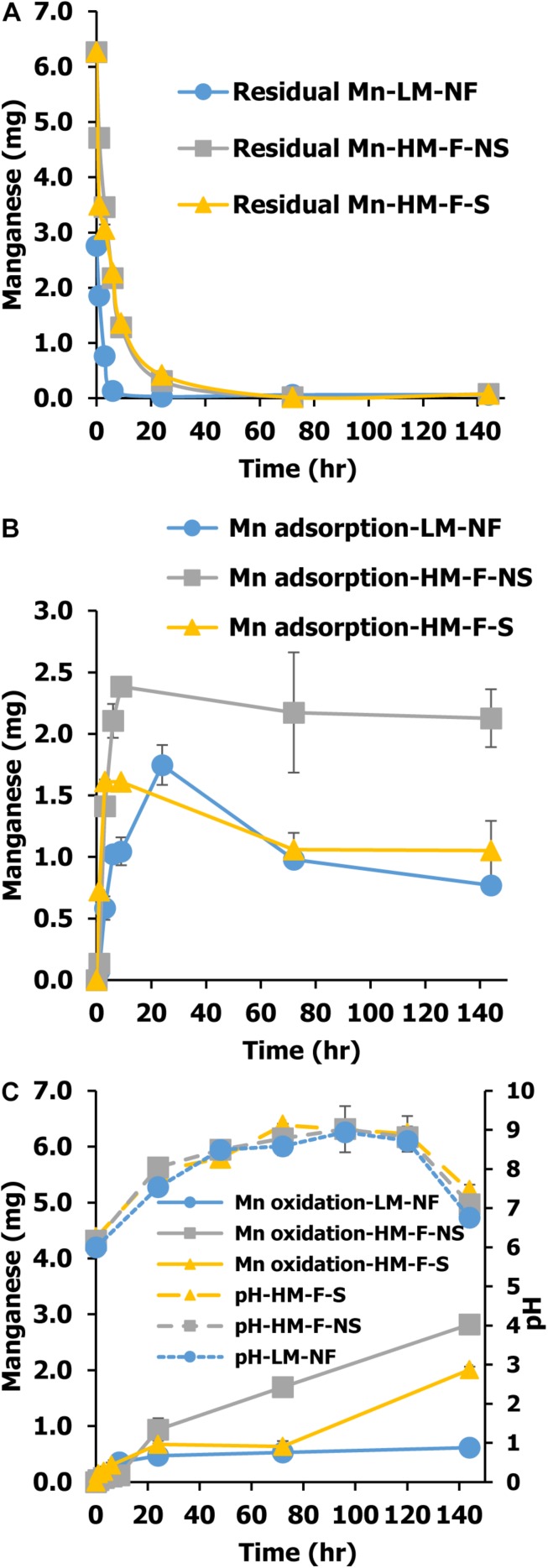
Mn residual **(A)**, Mn adsorption **(B)**, and Mn oxidation **(C)** of *Pediastrum duplex* AARLG060 cultivation using modified medium in LM-NF, HM-F-NS, and HM-F-S. LM-NF, low Mn non-filter; HM-F-NS, high Mn filter non-sterile; and HM-F-S, high Mn filter sterile.

Green microalga can be used to remediate Mn from the environment in two ways. In the direct process, the microalgae were taken up by the cells and in the indirect process, remediation occurred via the oxidation of Mn ion to Mn oxides. The variations of Mn remediation in each process involving the residual Mn and Mn adsorption values are shown in Figure 2. The residual Mn was found to rapidly decrease in all treatments. The fastest level of Mn remediation was observed in the low Mn treatment (LM-NF) at 9 h, while the high Mn treatments (HM-F-NS and HM-F-S) completed remediation with residual Mn after 12 h (Figure 3A). This outcome was in contrast to the degree of Mn adsorption that occurred. Microalga adsorbed Mn by binding the Mn ion to the cell surface. The adsorption values of all treatments revealed a similar inclination. The level of adsorption increased promptly within 24 h. At which point, the adsorption rate steadily and slightly declined (Figure 3B). However, the maximum level of adsorption was recorded in HM-F-NS, followed by HM-F-S and LM-NF, respectively. Another process involving Mn remediation that was observed in this experiment was Mn oxidation. The oxide form of the Mn (MnO*_*x*_*) was achieved when the pH level was increased to over 7.5 after 24 h (Figure 3C). The pH level in these treatments was increased from 6.00 on Day 0 to 7.54 after 24 h, and to over 7.5 after 120 h of the remediation process. The development of the Mn oxide yield increased laterally when the pH level was accelerated, especially in the high Mn treatments (HM-F-NS and HM-F-S). However, the degree of oxidation in the low Mn treatments was stable after 9 h.

### Mn Remediation Efficiency, Kinetics, and Isotherm Adsorption

**FIGURE 4:**
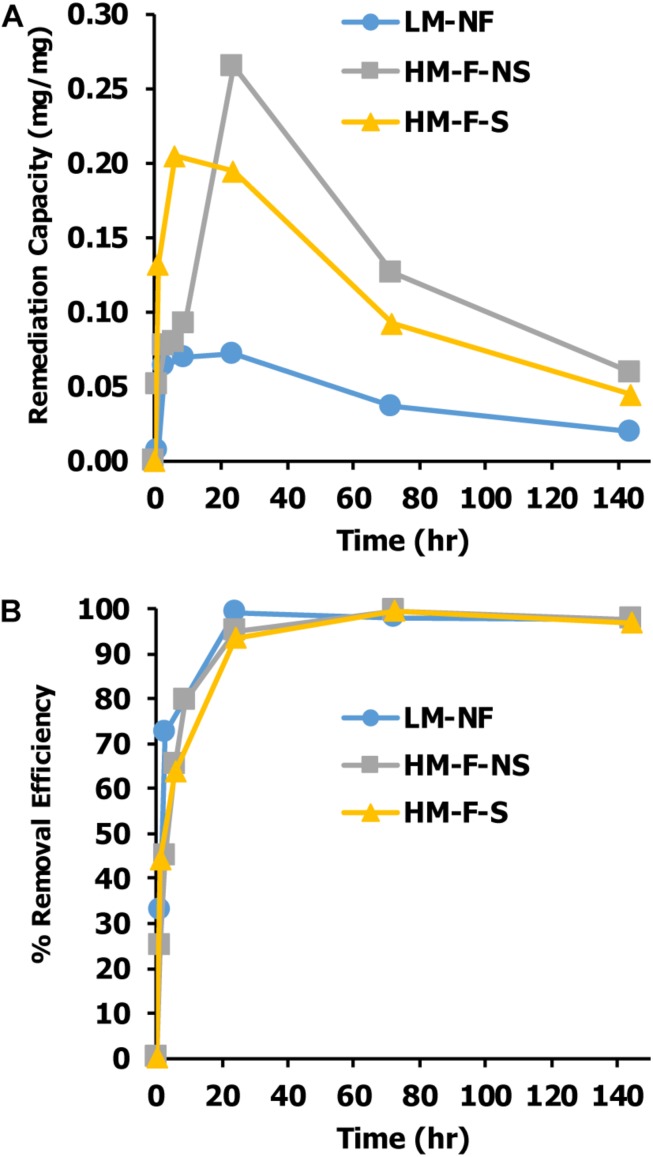
Effect of contact time on **(A)** Mn remediation capacity of *Pediastrum duplex* AARLG060 and **(B)** percent of Mn remediate efficiency. LM-NF, low Mn non-filter; HM-F-NS, high Mn filter non-sterile; and HM-F-S, high Mn filter sterile.

Capacities of Mn remediation were studied at different Mn concentrations and under different conditions. The results revealed that Mn concentrations in the aqueous phase decreased with time. After the period of equilibrium had passed, the Mn remediation capacity gradually declined. Remediation capacity was affected by the variations of Mn concentrations. In LM-NF, the equilibrium of microalgal Mn that was taken up from the solution was recorded at 0.0719 mg/mg at 24 h. Meanwhile, in the high Mn concentration treatments, the equilibrium of Mn uptake was recorded at 0.2646 mg/mg at 24 h (HM-F-NS) and 0.2049 mg/mg at 6 h (HM-F-S) (Figure 4A).

Percent removal efficiency is considered as the percentage of Mn that alga cultures take up from the solution. The results found that the level of percent of removal efficiency of all treatments rapidly increased after the first 3 h and achieved nearly 100% after 12 h of exposure time (Figure 4B).

**FIGURE 5:**
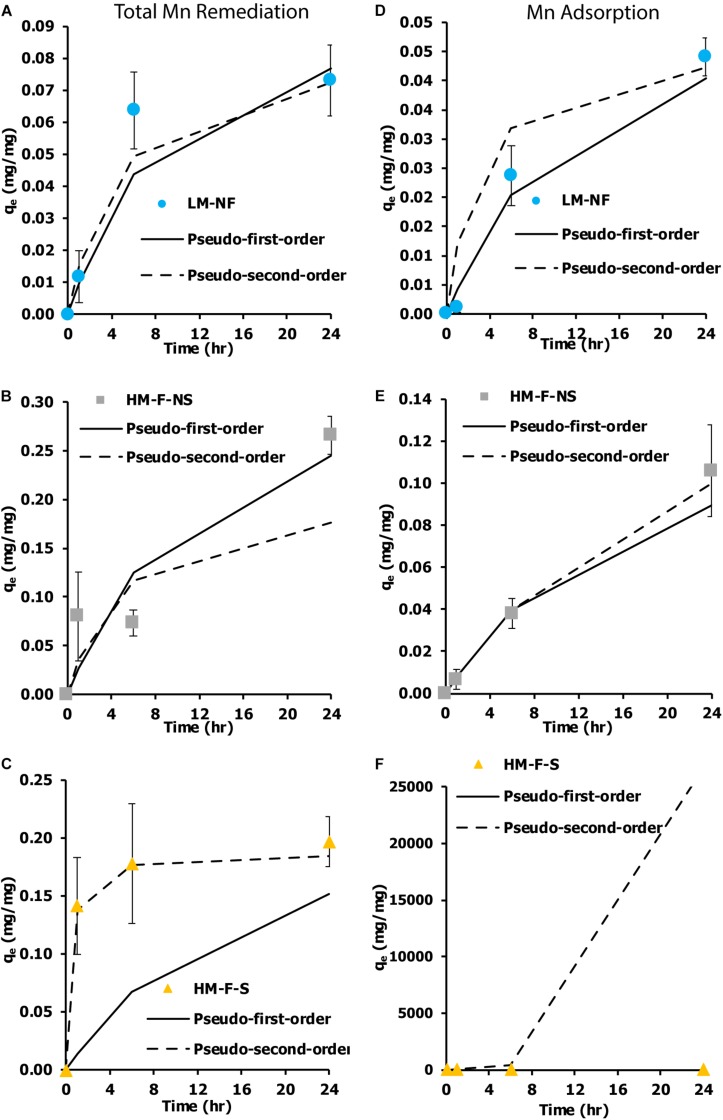
Kinetic measurement of Mn removal from the living microalgae *Pediastrum duplex* AARLG060 at various Mn concentrations and under various conditions. **(A–C)** Kinetic measurement of total Mn remediation in LM-NF, HM-F-NS, and HM-F-S. **(D–F)** Kinetic measurement of Mn adsorption in LM-NF, HM-F-NS, and HM-F-S. LM-NF, low Mn non-filter; HM-F-NS, high Mn filter non-sterile; and HM-F-S, high Mn filter sterile.

The kinetic measurement of Mn remediation was calculated from two values. First, the kinetic measurement of remediation was calculated from the residual Mn in the solution. This kinetic measurement was used to indicate the total Mn remediation of the microalga (adsorption and oxidation). Another significant value was the kinetic measurement of biosorption. This was calculated from Mn adsorption capacity. The kinetic measurement was determined within 24 h of contact time, which was the time that was needed to reach equilibrium. Two kinetic models were employed as a pseudo first-order and a pseudo second-order model based on the remediation capacity and the bio-sorption capacity of the biomass. These results were then compared. Figures 5A,C show the kinetic measurements of the Mn total remediation of LM-NF and HM-F-S that were observed following the non-linear graph of the pseudo second-order models. The HM-F-NS (Figure 5B) tendency of the graph presence kinetic model follows the pseudo first order and pseudo second order. With regard to the measurement of Mn adsorption, the non-linear graph tended to be in approximate agreement with the pseudo first-order model (Figures 5D–F).

**FIGURE 6:**
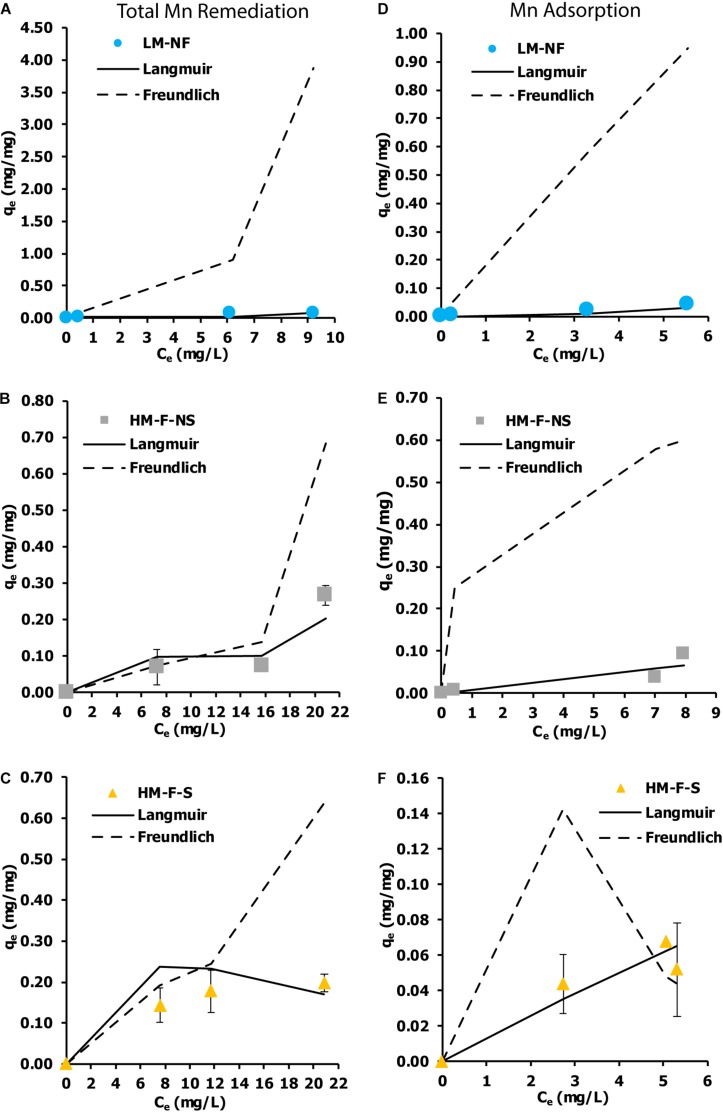
Biosorption isotherm of Mn by microalga *Pediastrum duplex* AARLG060. **(A–C)** Isotherm of total Mn remediation in LM-NF, HM-F-NS, and HM-F-S. **(D–F)** Isotherm of Mn adsorption in LM-NF, HM-F-NS, and HM-F-S. LM-NF, low Mn non-filter; HM-F-NS, high Mn filter non-sterile; and HM-F-S, high Mn filter sterile.

An isotherm indicates the distribution of the adsorbate between the liquid and solid phases when the adsorption process extends to a state of equilibrium. The Langmuir and Freundlich isotherm models are the most applied isotherm models used to determine the equilibrium of adsorption systems. The current results show that the remediation isotherm of LM-NF, HM-F-NS, and HM-F-S corresponded better to the Langmuir model than the Freundlich model (Figure 6). Langmuir isotherms reveal the characterization of mono-layer adsorption and are used to estimate the maximum metal uptake values within the adsorbent (*q*_*m*_). In this study, the maximum qm of the total Mn remediation isotherm was recorded in HM-F-S at 1.7391 mg/mg, and the level of adsorption in HM-F-S was recorded at 0.7052 mg/mg.

### Characterization of Bio-Mn Oxide

**FIGURE 7:**
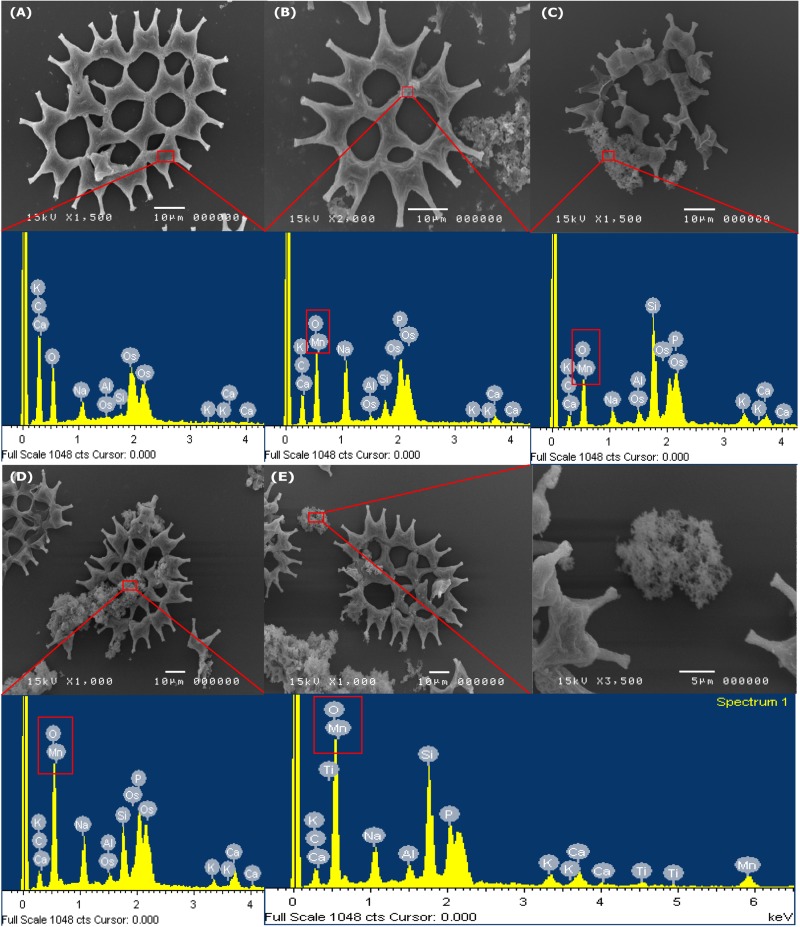
SEM images and EDS analysis of the MnO*_*x*_* on surface of *Pediastrum Duplex* AARLG060. **(A)** Microalga cell in culture on Day 0 and EDS spectra of selected area in surface cell. **(B–D)** Microalga cell and aggregated Mn particles in culture and EDS spectra of selected area on Day 6. **(E)** Aggregated Mn particles and EDS spectra of selected areas. LM-NF, low Mn non-filter; HM-F-NS, high Mn filter non-sterile; and HM-F-S, high Mn filter sterile.

A SEM is an instrument that is used to observe the Mn dioxide on the microalgae cell surface. The SEM images of LM-NF, HM-F-NS, and HM-F-S are presented in Figure 7. The results reveal that on the initial day (Figure 7A), the cells of microalga *P. duplex* AARLG060 were observed to be smooth without perforation and interstices between the cells. After 6 days of Mn remediation, the microalga cultures appeared brown as a result of the presence of Mn oxide. The granules of Mn complexes were observed on the microalga surface cells in the SEM images (Figures 7B–E). The microalga cell surface was covered with various sizes and irregular forms of MnO*_*x*_*. The relative elemental contents of Mn particles on the microalgal surface were investigated using energy-dispersive X-ray spectroscopy (EDS). The EDS spectrum of microalga from Day 0 indicated the absence of Mn ion on the microalgal surface. Mn was then detected in the EDS spectrum after Mn remediation on Day 6. The granules of Mn particles consisted of Mn and O in the complexes.

**FIGURE 8:**
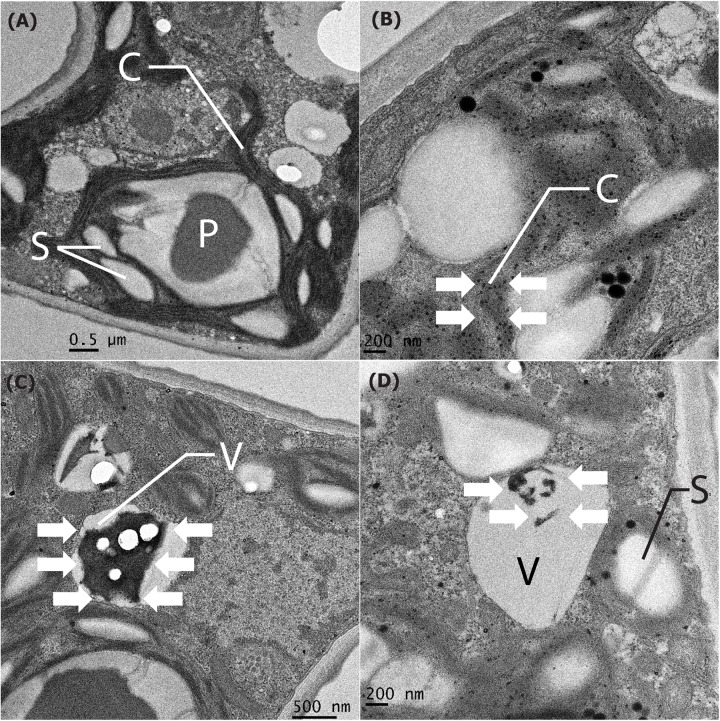
Cell compartment of *Pediastrum duplex* AARLG060 before/after Mn remediation. **(A)** Cell compartment of microalga before Mn remediation on Day 0. **(B–D)** The nano-size particles localized in the cell compartment of microalga (white arrows) after Mn remediation of LM-NF, HM-F-NS, and HM-F-S treatment on Day 6. C, chloroplast; P, pyrenoid; S, starch granules; V, vacuole; LM-NF, low Mn non-filter; HM-F-NS, high Mn filter non-sterile; and HM-F-S, high Mn filter sterile.

Manganese particles that were localized intracellularly were investigated using a TEM. On the first day of the experiment (Figure 8A), the intracellular compartments of the chloroplast and vacuoles were clear and presented as a small particle scatter. In addition, after Mn remediation on the 6th day, the ultrastructural alteration of *P. duplex* AARLG060 was observed. In LM-NF (Figure 8B), the small particles were scattered over the chloroplast. Meanwhile, the microalga ultrastructure of the HM-F-NS and HM-F-S treatments displayed nano-size particles that were localized in the vacuoles (Figures 8C,D). However, in both the HM-F-NS and HM-F-S treatments, small dense particles were not found to be present in the chloroplast.

## Discussion

Nutrients, such as nitrogen, phosphorus, and carbon, are classified as macronutrients and are required for algal growth (Encarnação et al., 2012). Based on the optical density (OD_665_) measurement, the OD_665_ value in all of the samples treated with modified medium increased rapidly, especially during the first 3 days. This indicates that the modified medium is suitable for microalgal cultivation, even under conditions of Mn contamination. The appropriate media composition with a suitable initial inoculum of OD_665_ at 0.2 could enhance the growth to a log phase without a lag phase. Notably, a shorter lag period will intensify the degree of remediation efficiency.

Interestingly, the uncontrollable factors in the natural wastewater did not affect the growth of microalgae. Filtration and sterilization steps were applied to the water collected from the rehabilitated reservoir. The main purpose of the filtered conditions used in this study was to remove colloidal particles and some microalgae. The removal of the colloidal particles may increase light penetration, thereby improving microalgal growth and some inorganic nutrient removal (Mayhead et al., 2018). In addition, the removal of the natural microalgae inhabiting this reservoir was done to reduce the inference of those indigenous microalgae on OD_665_ and Mn remediation. Notably, the purpose of the sterilized conditions was to eliminate other microorganisms that could present an interference in the experiment. Currently, the results have shown that the microalga *P. duplex* AARLG060 could be adapted to grow in natural wastewater collected from the habilitated reservoir. LM-NF has shown a trend of OD_665_ that is similar to the HM-F-NS and HM-F-S treatments. Events for the non-sterile and sterile conditions reveal that the growth of *P. duplex* AARLG060 in the HM-F-S treatment was higher than that of HM-F-NS. From this result, bacterial communities that are present in water samples could utilize the inorganic nutrients that would otherwise not be available for microalga. Previous studies have revealed that some natural bacteria may slightly inhibit microalgal growth (Segev et al., 2016). In addition, Mn concentrations may affect microalgal growth. The specific growth rate of the LM-NF treatment was calculated at 0.06 day^–1^, which was higher those that of the HM-F-NS and HM-F-S treatments (0.05 and 0.048 day^–1^, respectively). Previous studies have described the effect of Mn concentration on microalgae growth. At a low Mn concentration of 0.1 mg/L, the microalgal strain *Tetraselmis marina* AC16-MESO could increase cell density after 72 h, while the cell density decreased when the Mn concentration was as high as 20 mg/L (Cameron et al., 2018).

In the meantime, the pH value of the microalgae culture is another important factor that affects microalgae growth and supports Mn oxidation (Richardson et al., 1988). The initial pH value of the rehabilitated reservoir was recorded at 3.42. After adding the modified medium, the pH value was found to be >6.00. After cultivation with microalgae, the pH value in all treatments increased to over 7.5. The pH level as an alkaline condition was reported to be >8.0, which is known to be an appropriate pH value for Mn oxidation (Richardson et al., 1988). Bio-oxidation was presented after Mn ions were adsorbed on the microalga cells, while the photosynthetic activity of the microalgae produced oxygen at the same time. These two phenomena encouraged oxygen to oxidize Mn ions resulting in Mn oxide, which appeared as dark brown solid particles. In addition, pH may also have affected the cell size and shape of *P. duplex.* Consequently, the microalgal morphology may be altered at different pH levels (Neustupa and Hodac, 2005), which may reflect the surface to volume ratio and affect both the adsorption potential and the relevant kinetics.

In the Mn remediation treatment under LM-NF, HM-F-NS, and HM-F-S conditions, the amount of residual Mn decreased depending on the initial concentration value and cultivation time. The results also revealed that living microalga *P. duplex* AARLG060 could remove Mn from the solution through a variety of processes. Mn ion may be removed from the solution by being bound with various functional groups that are present on the cell wall (Kaplan, 2013).

Another important process is that of bio-oxidation. Microalgae can generate Mn oxide under alkaline conditions (Richardson et al., 1988). At low levels of Mn concentrations (LM-NF), Mn was removed over a period of 0–24 h. Meanwhile, at higher concentrations, HM-F-NS and HM-F-S required longer amounts of time to remove the residual Mn. High amounts of Mn can affect the Mn remediation process (Abbas et al., 2014). For the biosorption process, HM-F-NS treatment revealed a higher adsorption level than that of HM-F-S according to the treatment time. Previous studies have suggested that in non-sterile treatments, the microalgal could be aggregated by polysaccharides or proteins that are produced from bacteria (Fuentes et al., 2016). This interaction could improve Mn biosorption within the treatment.

In comparisons with other adsorption processes, most researchers used dried algal biomass for which the remediation was based on the organic/inorganic sorbent. However, the chemical interaction between the sorbent and adsorbent was limited by the adsorption capacity, which remained constant after reaching equilibrium. The advantages of using living microalga include the variations of remediation capacity. In this research study, a low Mn concentration of 9 mg/L may be the boundary level of the remediation capacity of this microalga, which could be indicated from nearly 100% removal and low amounts of the product obtained from Mn oxidation. However, at high Mn concentrations, even the degree of adsorption reached equilibrium after 24 h. However, Mn oxidation was still prolonged and tended to increase over the time. Thus, the results indicate that living microalga may reveal a higher level of remediation capacity for high Mn concentrations than other non-living chemical interaction adsorbents.

The oxide form of Mn oxidation (MnO*_*x*_*) occurred when the residual Mn diminished and the pH level increased. Previous authors have suggested that high pH levels are the main factors for Mn oxidation (Richardson and Stolzenbach, 1995; Wang et al., 2017). The present study demonstrated that the microalga *P. duplex* AARLG060 generated a portion of MnO*_*x*_* on the 6th day of exposure. Similarly, previous studies have reported that biogenic Mn oxides can be generated by *Desmodesmus* sp. WR1 from an initial Mn concentration of 6–30 mg/L (Wang et al., 2017). The differing amounts of Mn oxidation could depend on the degree of oxygen production, the existing pH value, the amount of microalgae present, and the growth of each microalgae species.

Initial Mn concentrations in aqueous solutions are strongly influenced by metal uptake in the adsorption of Mn ions. The adsorption capacity of the microalga in the HM-F-NS and HM-F-S treatments was higher than that of the LM-NF treatment. These results were in accordance with those of the previous study, which revealed that high concentrations of Mn in the solution could interact more within the active site (Ghoneim et al., 2014).

According to the results of LM-NF and HM-F-S, part of the total Mn remediation was represented by a characteristic of the pseudo second-order model, which was demonstrated as a characteristic of Mn adsorbed reaction based on the sorption capacity of the microalgal cells at the solid phase (chemisorption). However, HM-F-NS was approximately representative of the pseudo first model. In addition, part of the Mn adsorption was presented as a characteristic of the pseudo first-order model. This indicates that Mn was remediated and adsorbed based on the solution concentration for the sorption of Mn from the liquid phase on the surface of the microalga cells (Ho and McKay, 1999). As compared to the findings reported in previously published papers, the remediation of lead using non-living microalgae that had been pretreated with CaCl_2_ revealed similar characteristics of the pseudo second order in this experiment (Hanbali et al., 2014). However, the results of remediation in this study did not completely correlate with those of the pseudo second order because the remediation kinetics were intervened by Mn oxidation.

Although some researchers have reported on isotherm adsorption of cadmium remediation using living microalga, *Phaeodactylum tricornutum* (Torres et al., 2014), to our knowledge, the isotherm adsorption of Mn from living microalga has not yet been investigated. The living microalga *P. duplex* AARLG060 used in this study demonstrated a potential for Mn remediation. Most of the remediation treatments were closely fitted to the Langmuir isotherms, for which the adsorption occurred at specific homogeneous sites and could be used to estimate the maximum metal uptake values within the adsorbent. The maximum adsorption capacities (*q*_*m*_) of this treatment were calculated as 1.7391 mg/mg. In a previously published paper on the biosorption of heavy metals using living green algae, it was reported that *Scenedesmus quadricauda* displayed a range of biosorption capacity between 3.54 and 75.20 mg/g (Kizilkaya et al., 2012). *Scenedesmus obliquus* was used to study the maximum adsorption capacity of iron (III) adsorption. In that study, the maximum adsorption capacity was recorded at 2.2 mg/g from the initial concentration iron (III) of 25 mg/L. In those reports, *P. duplex* AARLG060 displayed lower levels of biosorption capacity than *S. quadricauda* and *S. obliquus.* This may be because the samples of *S. quadricauda* and *S. obliquus* used in those studies were considered high with regard to the degree of cell density within the culture. Notably, the adsorption capacities were dependent upon the cell number, which increased the cellular surface and enhanced the degree of adsorption.

The SEM illustrated the degree of precipitation of Mn on the microalga cell surface. EDS analysis was used to confirm that the dark brown particles consisted of Mn and oxygen. According to previous studies, bio-MnO*_*x*_* could be produced by green microalgae. During microalgae growth, the microalgae took up CO_2_ and produced oxygen, which could have increased pH levels. This is important for the oxidation of Mn to MnO*_*x*_* (Greene and Madgwick, 1991; Knauer et al., 1999; Wang et al., 2017; Thongpitak et al., 2018). This phenomenon was also recorded in studies of other microalgae such as *Chlorella* sp., *Desmodesmus* sp. WR1, and *Scenedesmus subspicatus* (Richardson et al., 1988; Knauer et al., 1999; Wang et al., 2017).

Moreover, when the Mn absorption process occurs, some soluble Mn ions could be transferred into the cell, become fixed with metalloid proteins, and accumulate within the vacuole of the microalgae (Mehta and Gaur, 2005; Kaplan, 2013). Although the precision method for the measurement of intracellular Mn has yet to be developed, the absorption of Mn may be perceived from the dissimilarity between the summary of adsorption and oxidation within the residual in the solution. In addition, the particulate accumulation of Mn deposits inside the cell compartment of living microalgae could be observed using the TEM technique (Shebanova et al., 2017). The ultrastructure of the *P. duplex* AARLG060 cells that had been treated with Mn revealed that the dark granules were scattered in the chloroplast where photosynthesis occurs. In the chloroplast, solar energy is converted into biochemical energy release O_2_ (Engel et al., 2015; Yang et al., 2017). Transition metals, such as Mn, play an essential role as redox active centers in photosynthetic electron transfer reactions, in which they serve to catalyze the actual oxidation of water (Renger and Wydrzynski, 1991). Naturally, microalgae use Mn in very low amounts in PS II. Thus, when microalgae take up high amounts of Mn in cultures, the remaining Mn in the chloroplast is oxidized to MnO*_*x*_* and localized in the chloroplast. Previous studies have reported similar results to those of this study. Iron, the essential trace element for chlorophyll, was found to be distributed in the chloroplast of the green microalga *Coccomyxa actinabiotis* (Leonardo et al., 2014). Toxic heavy metals, such as silver, were also found to be mainly present in the chloroplast and mitochondria. This indicates that the chloroplast contains enzymes that are associated with nanoparticle formation (Leonardo et al., 2016). The dark granular precipitate was also found to be localized in the vacuole. The results indicate that metals accumulated in the plant. Similar results from studies on the contaminant depositions in plant biomass were reported in terms of other uptake studies of lead by *Typha angustifolia* (Panich-pat et al., 2005).

This can be described as a common mechanism for intracellular metal detoxification in living microalgae obtained from the formation of metal-binding proteins such as metallothioneins (Kaplan, 2013). Previous studies have reported that the metal deposition on the ultrastructure of microalgae was associated with the process of heavy metal remediation. The effects of heavy metals on the algal ultrastructure were dependent upon concentration values and cultivation time. Previous studies have reported on the impacts of chromium on the unicellular model system. An increase in the number of starch grains and the degree of precipitation of chromium was found in some parts of the cell wall of *Micrasterias denticulata* (Volland et al., 2012). The vacuole is the main part of the structure involved in detoxifying and storing heavy metals (Kaplan, 2013; Shebanova et al., 2017). An increase in the vacuolar deposits in the cells of *Chlamydomonas acidophila* that had been treated with a large amount of cadmium was observed (Nishikawa et al., 2003). Similarly, cadmium was also found to have accumulated within the vacuole of the marine diatom *Skeletonema costatum* (Nassiri et al., 1997). Many researchers have previously reported that electron dense deposits acquired from heavy metals were localized in the vacuole and assisted in protecting microalgae cells from metal toxicity.

## Conclusion

In conclusion, the living microalga *P. duplex* AARLG060 was found to be a potent strain for Mn remediation. This strain could be grown in the natural wastewater collected from a rehabilitated lignite coal mine reservoir without any effects in terms of the biotic or abiotic factors present in the water. In addition, high Mn concentrations of up to 20 mg/L could be rapidly remediated within 24 h. The remediation of the living microalga could be performed from many processes such as via biosorption, bio-oxidation, and absorption. The information attained in this study will be highly beneficial for outdoor large-scale Mn remediation in the future.

## Data Availability Statement

The datasets analyzed in this manuscript are not publicly available. Requests to access the datasets should be directed to mailto:chayakorn.pumas@gmail.com.

## Author Contributions

All authors listed have made a substantial, direct and intellectual contribution to the work, and approved it for publication.

## Conflict of Interest

The authors declare that the research was conducted in the absence of any commercial or financial relationships that could be construed as a potential conflict of interest.
