# Supplementary material for: Remediation of Manganese-Contaminated Coal-Mine Water Using Bio-Sorption and Bio-Oxidation by the Microalga Pediastrum duplex (AARLG060): A Laboratory-Scale Feasibility Study
Source: Front Microbiol. 2019 Nov 12;10:2605. doi: 10.3389/fmicb.2019.02605 (PMC6861300; doi:10.3389/fmicb.2019.02605)
Supplement: Supplementary file 1 [file Data_Sheet_1.PDF]

SUPPLEMENTARY MATERIALS

Remediation of Manganese-Contaminated Coal-Mine Water Using Bio-Sorption and Bio-Oxidation by the Microalga *Pediastrum duplex* (AARLG060): A Laboratory-Scale Feasibility Study

Jakkapong Thongpitak[1], Jeeraporn Pekkoh[2], Chayakorn Pumas[2*]

[1]PhD Degree Program in Environmental Science, Environmental Science Research Center, Faculty of Science, Chiang Mai University, Chiang Mai, 50200, Thailand

[2]Center of Excellence in Bioresources for Agriculture, Industry and Medicine, Chiang Mai University, Department of Biology, Faculty of Science, Chiang Mai, 50200, Thailand

Table 1: The amount of dry biomass used in the sorption calculation.

Microalga dry weight					
LM-NF		HM-F-NS		HM-F-S	
Time (hr)	Weight (mg)	Time (hr)	Weight (mg)	Time (hr)	Weight (mg)
0	15.00	0	27.00	0	9.00
0	13.00	0	22.50	0	12.00
0	18.00	0	18.00	0	10.50
AVG	15.33	AVG	22.50	AVG	10.50
SD	2.51	SD	4.50	SD	1.50
1	216.00	1	48.00	1	15.00
1	42.00	1	12.00	1	27.00
1	129.00	1	30.00	1	21.00
AVG	129.00	AVG	30.00	AVG	21.00
SD	87.00	SD	18.00	SD	6.00
6	35.00	6	54.00	6	33.00
6	36.00	6	69.00	6	21.00
6	51.00	6	48.00	6	18.00
AVG	40.66	AVG	57.00	AVG	24.00
SD	8.96	SD	10.81	SD	7.93
24	45.00	24	21.00	24	27.00
24	36.00	24	24.00	24	33.00
24	33.00	24	22.50	24	30.00
AVG	38.00	AVG	22.50	AVG	30.00
SD	6.24	SD	1.50	SD	3.00
